# Large ciliary body melanocytoma with pseudocysts: a case report

**DOI:** 10.1186/s12886-023-02778-8

**Published:** 2023-01-17

**Authors:** Allie Simpson, Nick Mamalis, Christopher Gee, Roger P. Harrie

**Affiliations:** 1 John A. Moran Eye Center, University of Utah, 65 N Mario Capecchi Dr, Salt Lake City, UT 84132 USA; 2 Salt Lake Retina, 3855 7800 S, #100, West Jordan, UT 84088 USA

**Keywords:** Melanocytoma, A-scan, Pseudocysts, Case report

## Abstract

**Background:**

To describe the correlation between standardized A-scan echography and histopathology in a ciliary body melanocytoma.

**Case presentation:**

We present a case of a large ciliary body melanocytoma with significant growth, vision loss, and elevated intraocular pressure that was diagnosed clinically as a melanoma, but the standardized A-scan findings correlated to the histopathological description of a melanocytoma with multiple pseudocysts.

**Conclusions:**

The reflectivity of this melanocytoma by standardized A-scan was consistent with multiple pseudocysts on pathological evaluation. This echographic pattern guided the differential diagnosis. Standardized A-scan is an important diagnostic tool in the differentiation of ciliary body melanocytomas from melanomas.

## Introduction

Choroidal and ciliary body melanocytomas are rare and may be confused with choroidal melanomas, particularly when rapid growth, large size, and elevated intraocular pressure are present. Most melanocytomas are under 2 mm in thickness which makes it difficult to evaluate internal reflectivity by ultrasound [1, 2]. However, standardized A-scan is an important diagnostic tool in the differentiation of ciliary body melanocytomas from melanomas [3].

## Case presentation

**Fig. 1:**
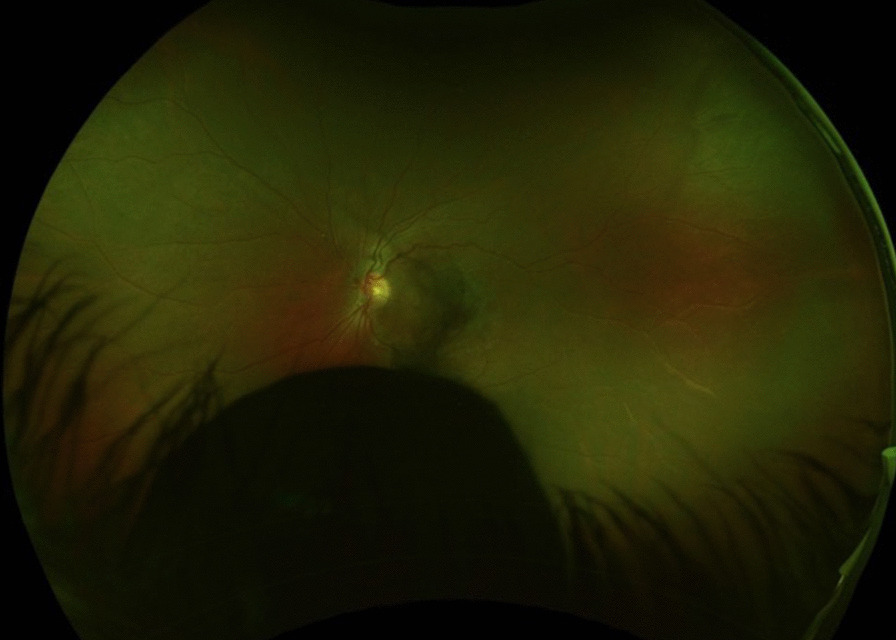
Fundus photo of the left eye showing a peripheral, darkly pigmented lesion

**Fig. 2:**
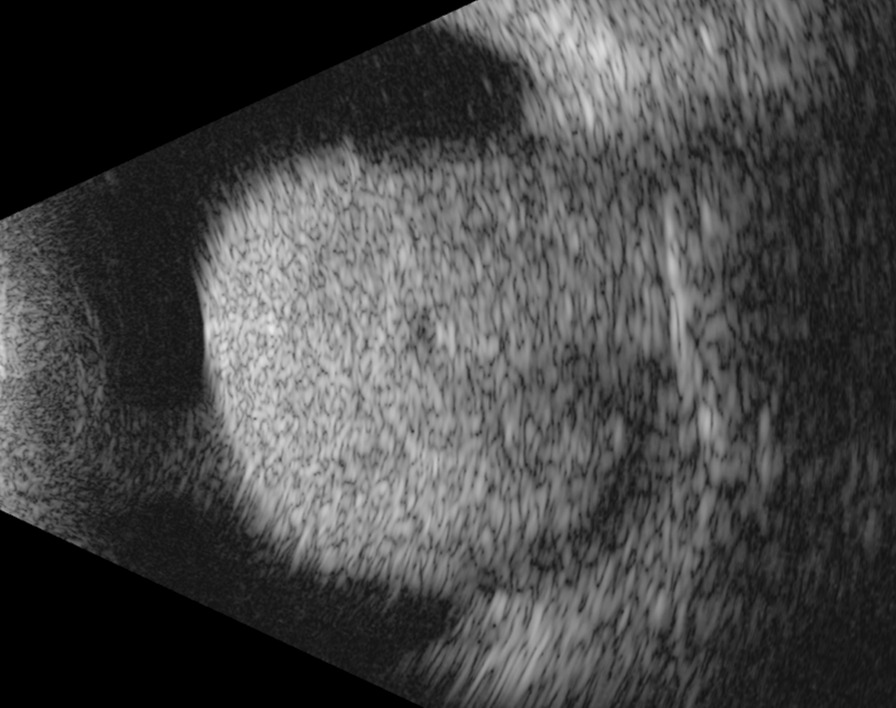
B-scan ultrasound exhibiting a large mushrooming mass of the ciliary body

**Fig. 3:**
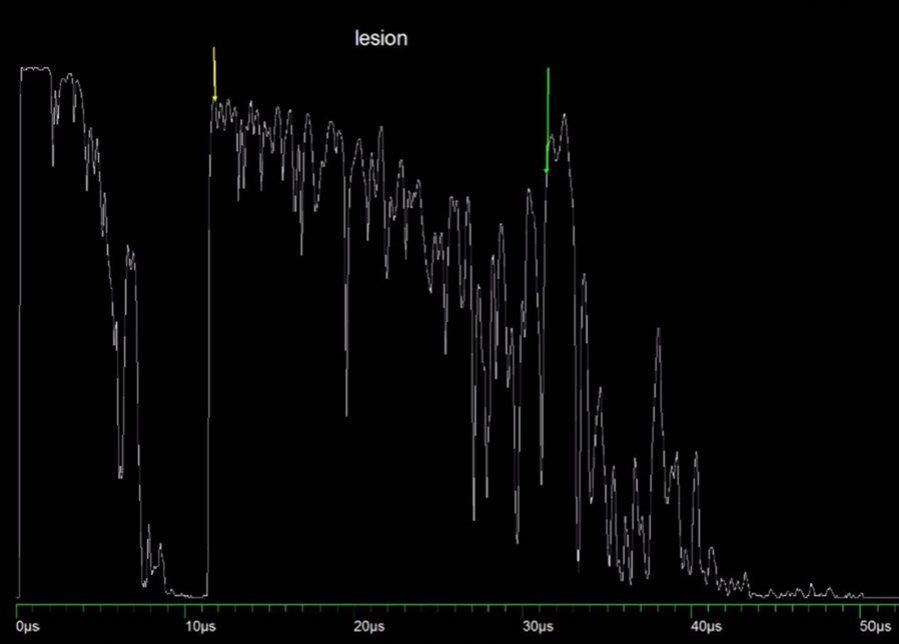
Standardized A-scan image of the highly reflective lesion with regular internal reflectivity

A 20-year-old woman presented with chronic floaters OS. Examination revealed visual acuity of 20/20 OD and 20/25 OS with intraocular pressures of 14 mm OU, and a normal slit-lamp examination OU. The right fundus examination was unremarkable, and the left revealed a peripheral, homogenously pigmented intraocular lesion inferiorly (Fig. 1). Ultrasonography showed a large mushrooming tumor on B-scan (Fig. 2) and a highly reflective lesion measuring 9.88 mm in thickness by 11.06 mm by 10.75 mm in basal dimensions on A-scan (Fig. 3). The reflectivity was not consistent with melanoma and the differential diagnosis included melanocytoma, adenoma of the pigmented ciliary body epithelium, and medulloepithelioma.

She was scheduled for a follow-up in 4 months but failed to keep several appointments and returned 3 ½ years later with left eye pain and loss of vision over several weeks. Examination at that time revealed a visual acuity of 20/20 OD and a small area of hand motion OS. Her intraocular pressures were 17 mm OD and 42 mm OS. Slit-lamp examination OS showed mild conjunctival injection without dilation of conjunctival vessels, mild corneal edema, and posterior synechiae with heavy deposition of pigment on the posterior lens capsule. There was no red reflex and no view of the fundus.

**Fig. 4:**
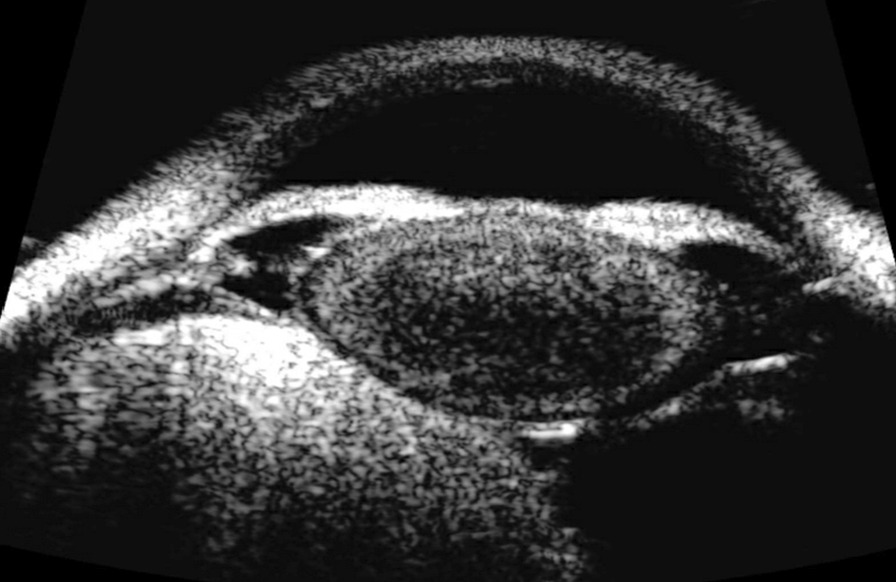
Ultrasound biomicroscopy showing an incompletely characterized lesion of ciliary body abutting the crystalline lens

**Fig. 5.**
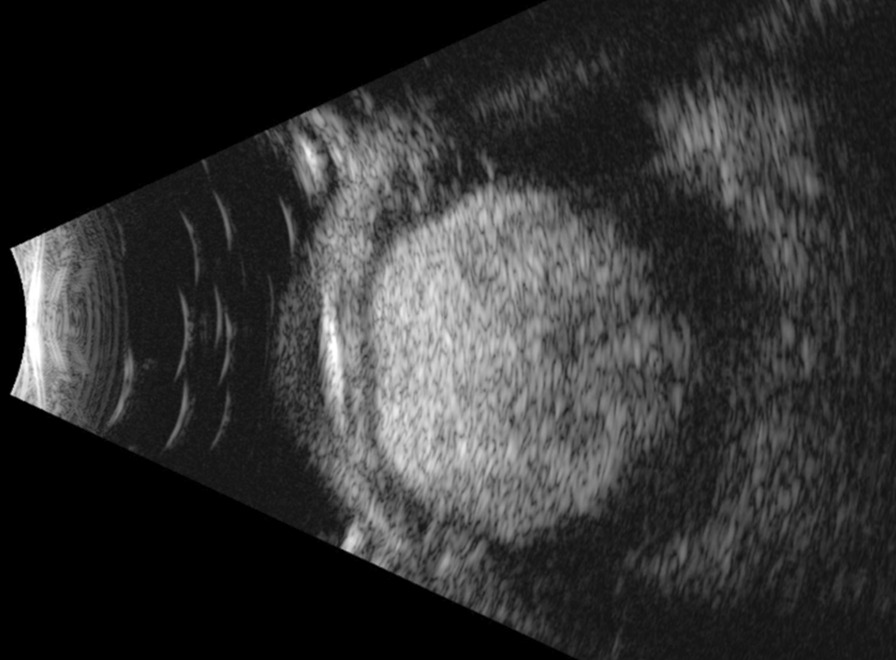
10 MHz immersion B-scan ultrasound displaying a large ciliary body lesion in its entirety

An ultrasound exam demonstrated a lesion measuring 15.73 mm in thickness by 16.43 mm by 15.94 mm in basal dimensions. Due to the size of the lesion, ultrasound biomicroscopy was unable to fully characterize the lesion (Fig. 4). The lesion was visualized in its entirety on 10 MHz immersion B-scan ultrasound (Fig. 5).

The significant growth and intraocular pigment deposition were concerning for a melanoma, but the high reflectivity on A-scan was most consistent with a melanocytoma or adenoma of the pigmented ciliary body epithelium. Due to the continued clinical concern for melanoma, despite the ultrasound findings, enucleation was proposed to the patient given the size of the lesion and status of the eye. Through shared decision-making, the patient elected for enucleation.

**Fig. 6:**
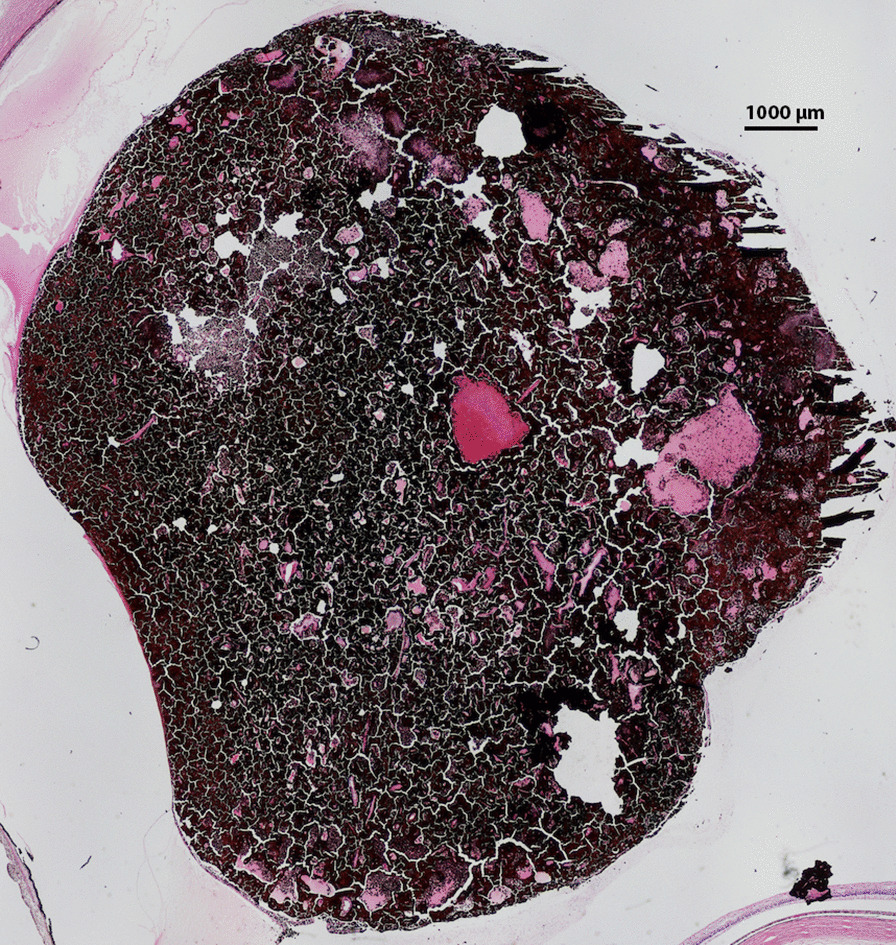
Light photomicrograph of six fields of view were combined to show a full view of the ciliary body mass with marked, dark pigmentation (hematoxylin and eosin stain; original magnification × 20). There were no color channel adjustments made to the merged image

**Fig. 7:**
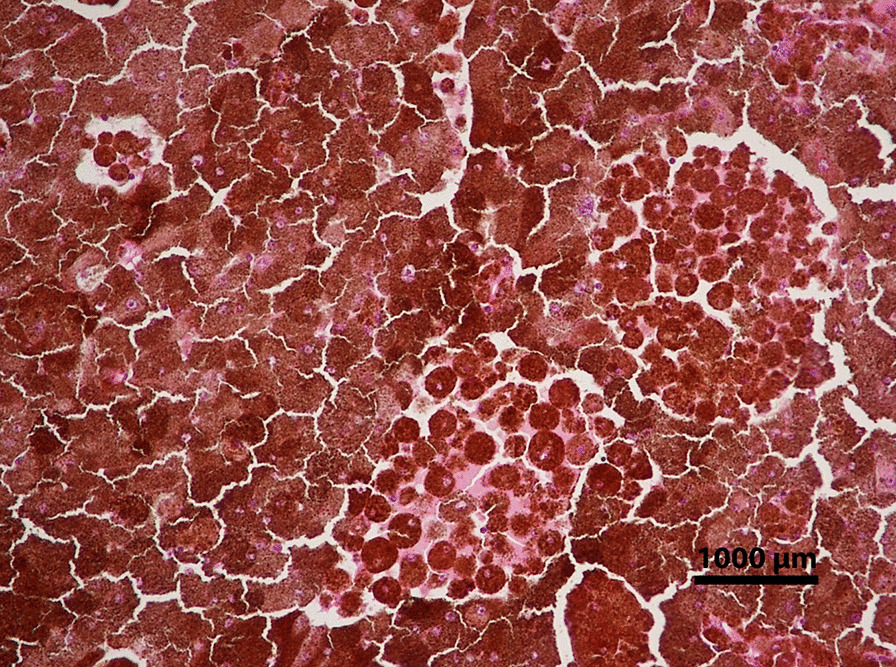
Light photomicrograph of a bleached histologic section obtained from the ciliary body mass exhibiting darkly pigmented cells (hematoxylin and eosin stain; original magnification × 200)

**Fig. 8:**
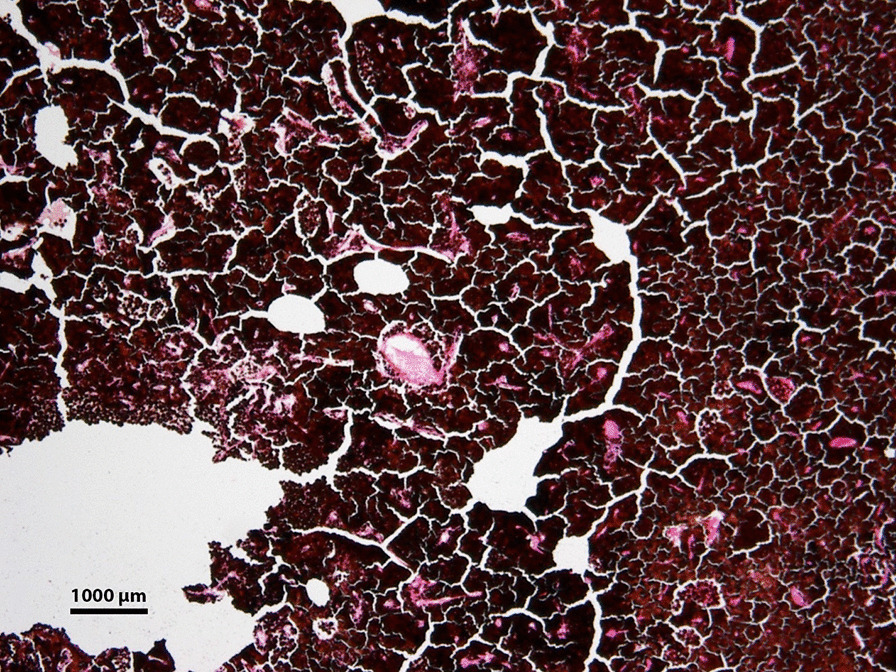
Light photomicrograph of a histologic section obtained from the ciliary body mass displaying pseudocyst formation (hematoxylin and eosin stain; original magnification × 100)

**Fig. 9:**
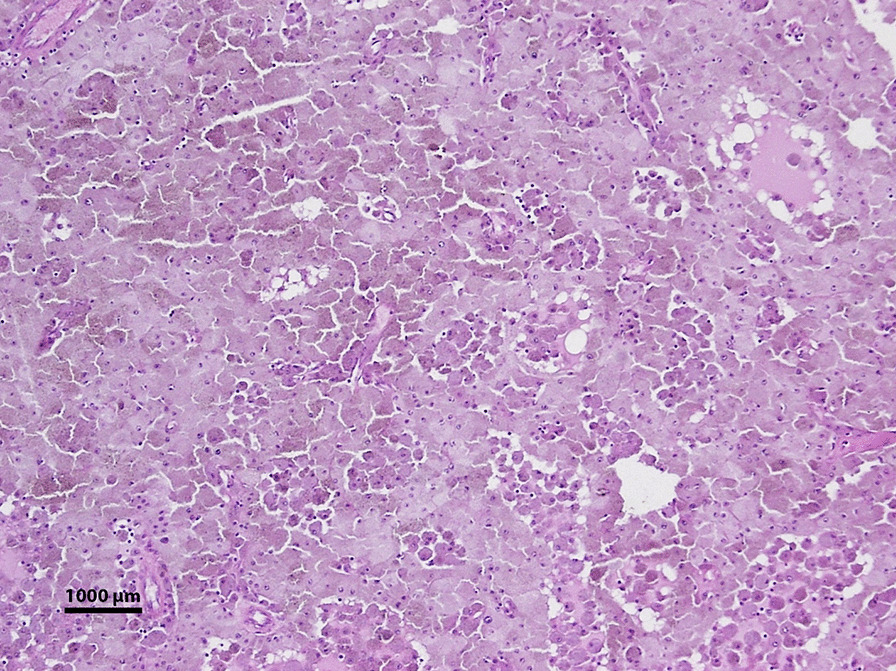
Light photomicrograph of a twice-bleached histologic section obtained from the ciliary body mass showing bland nuclei and low nucleus-to-cytoplasm ratio (hematoxylin and eosin stain; original magnification × 100)

The eye was enucleated, and pathology described a large ciliary body mass. Low power microscopy revealed a melanocytoma with pseudocysts (Fig. 6). Higher power microscopy showed cells with heavily pigmented cytoplasm, small nuclei without dysplastic features, and pigment-laden macrophages (Fig. 7). The pseudocyst formation was demonstrated (Fig. 8). Microscopy of double-bleached slides revealed bland nuclei, small nucleoli, and low nucleus-to-cytoplasm ratio typical of melanocytomas (Fig. 9). The microscope used for pathology imaging was an Olympus model BX40F4 with Olympus DP20 mounted camera. No detectors, filters, or acquisition software were utilized.

## Discussion and conclusions

Choroidal and ciliary body melanocytomas are rare and may be confused with choroidal melanomas especially when large in size. Other features including significant growth, pain, and increased intraocular pressure can lead the clinician to the misdiagnosis of malignant melanoma. Most melanocytomas are under 2 mm in thickness which makes it difficult to evaluate internal reflectivity by ultrasound [1]. This difficulty is reflected by some authors stating that most melanocytomas are low reflective and others that they are usually high reflective [2, 4]. The internal structure of intraocular tumors is best evaluated by standardized A-scan and is directly correlated to the histologic architecture of lesions [1, 3].

Prior to the routine use of ultrasonography, the misdiagnosis of choroidal melanomas was as high as 20% utilizing indirect ophthalmoscopy and fluorescein angiography [5]. The Collaborative Ocular Melanoma Study included the standardized A-scan as its primary ancillary testing modality with a resultant diagnostic accuracy of over 99% [6]. Densely cellular lesions such as melanomas with a relatively homogenous structure are low reflective because of the relative lack of interfaces for reflection of the sound beam. Conversely, lesions such as choroidal hemangiomas are high reflective because of the multiple interfaces due to the uniform arrangement of cystic blood-filled spaces. Robertson et al. described the case of a melanocytoma that was felt clinically to be a choroidal melanoma based on the appearance of a pigmented lesion with a collar-button configuration. It had low and regular internal reflectivity on A-scan which correlated histopathologically to a densely cellular tumor without the echographic interfaces created by necrosis or pseudocysts [7].

In the present case of a melanocytoma, the microscopic sections showed multiple pseudocysts which were reflected by the incident ultrasound beams to cause high reflectivity on the A-scan. To our knowledge this is the only report to demonstrate the correlation between the histopathology of a large melanocytoma with pseudocysts and the A-scan findings of high reflectivity. The ophthalmoscopic appearance of a darkly pigmented tumor was not consistent with a hemangioma and despite a mushrooming appearance on B-scan, the high reflectivity on A-scan was against the diagnosis of a choroidal melanoma. These findings supported the diagnosis of melanocytoma.

## Data Availability

All the data supporting our findings is contained within the manuscript.

## Abbreviations

OD: Right eye
OS: Left eye
OU: Both eyes
